# Macrophage cytokine responses to commensal Gram-positive *Lactobacillus salivarius* strains are TLR2-independent and Myd88-dependent

**DOI:** 10.1038/s41598-021-85347-7

**Published:** 2021-03-15

**Authors:** Sreeram Udayan, Ludovica F. Buttó, Valerio Rossini, Janaki Velmurugan, Maria Martinez-Lopez, David Sancho, Silvia Melgar, Paul W. O’Toole, Ken Nally

**Affiliations:** 1 APC Microbiome Ireland, University College Cork, Cork, Ireland; 2 School of Biochemistry and Cell Biology, University College Cork, Cork, Ireland; 3 Immunobiology Laboratory, Centro Nacional de Investigaciones Cardiovasculares (CNIC), Madrid, Spain; 4 School of Microbiology, University College Cork, Cork, Ireland

**Keywords:** Immunology, Microbiology

## Abstract

The mechanisms through which cells of the host innate immune system distinguish commensal bacteria from pathogens are currently unclear. Toll-like receptors (TLRs) are a class of pattern recognition receptors (PRRs) expressed by host cells which recognize microbe-associated molecular patterns (MAMPs) common to both commensal and pathogenic bacteria. Of the different TLRs, TLR2/6 recognize bacterial lipopeptides and trigger cytokines responses, especially to Gram-positive and Gram-negative pathogens. We report here that TLR2 is dispensable for triggering macrophage cytokine responses to different strains of the Gram-positive commensal bacterial species *Lactobacillus salivarius*. The *L. salivarius* UCC118 strain strongly upregulated expression of the PRRs, Mincle (*Clec4e*), TLR1 and TLR2 in macrophages while downregulating other TLR pathways. Cytokine responses triggered by *L. salivarius* UCC118 were predominantly TLR2-independent but MyD88-dependent. However, macrophage cytokine responses triggered by another Gram-positive commensal bacteria, *Bifidobacterium breve* UCC2003 were predominantly TLR2-dependent. Thus, we report a differential requirement for TLR2-dependency in triggering macrophage cytokine responses to different commensal Gram-positive bacteria. Furthermore, TNF-α responses to the TLR2 ligand FSL-1 and *L. salivarius* UCC118 were partially Mincle-dependent suggesting that PRR pathways such as Mincle contribute to the recognition of MAMPs on distinct Gram-positive commensal bacteria. Ultimately, integration of signals from these different PRR pathways and other MyD88-dependent pathways may determine immune responses to commensal bacteria at the host-microbe interface.

## Introduction

*Lactobacillus salivarius* (*L. salivarius*) is a widely studied Gram-positive commensal bacteria of the phylum Firmicutes, one of the dominant phyla of the human gut microbiome^1,2^. Recent research has focused on understanding the beneficial effects of *L. salivarius* as a candidate probiotic because of its ability to induce tolerogenic T cell responses^3,4^, ameliorate colitis^5^, translocate from the maternal gut to breast milk^6^, modulate immune-related functions of host intestinal epithelial cells^7,8^, induce antimicrobial activity^9,10^, induce anti-tumour activity^11^, maintain gastro-intestinal barrier integrity^12^ and induce anti-inflammatory activity^7,13^. While some of these properties have been attributed to the production of a well characterized bacteriocin Abp118 (an antibacterial peptide), the host signaling pathways required for mediating recognition and responses to *L. salivarius* are poorly characterised^10,14,15^. The interaction and effects of *L. salivarius* on intestinal epithelial cells were previously reported by our group^16^, but little is known about the mechanisms underpinning *L. salivarius* recognition by cells of the innate immune system such as macrophages. MAMPs (microbe-associated molecular patterns) on bacteria are recognized by pattern recognition receptors (PRRs) on host cells and this interaction plays a critical role in shaping microbe-specific innate and adaptive immune responses^17,18^. TLRs are an important class of PRRs which are involved in bacterial recognition. Upon binding their respective ligands, TLRs (such as TLR1, 2, 4 and 6) interact with adaptor molecule MAL (myelin and lymphocyte protein). TLRs (such as TLR1, 2, 4, 5, 6, 7 and 9) interact with MyD88 (myeloid differentiation primary response 88). TLR4 interacts with TRAM (translocating chain associated membrane protein) and other TLRs (such as TLR3 and TLR4) interact with TRIF (TIR-domain-containing adapter-inducing interferon-β). The interaction of TLRs with these different adaptor molecules ultimately initiate signaling cascades culminating in the activation of transcription factors such as NF-κB (nuclear factor kappa-light-chain-enhancer of activated B cells), AP-1 (activator protein 1) and IRFs (interferon regulatory factors)^19^. These in turn induce transcription of pro-inflammatory cytokines such as interleukin 6 (IL-6), tumour necrosis factor alpha (TNF-α), IL-12, IL-1β, anti-inflammatory cytokines such as IL-10 and chemokines like KC, type I interferons (IFNs), anti-microbial and anti-viral genes^17,20^.

The consequences of MAMP-PRR recognition are diverse and largely dependent on the molecular and biochemical characteristics of both the bacterial and host cells which interact with one another^21^. When hosts are infected with pathogens such as *Listeria monocytogenes*, *Porphyromonas gingivalis* and *Brucella microti,* recognition of their respective MAMPs by TLRs trigger inflammatory responses that aid in their clearance and are thus protective to the host^22–24^. However, other pathogens such as *Salmonella typhimurium* require initial MAMP-TLR recognition for inducing production of specific virulence factors that provide them with a survival benefit by evading host immune surveillance^25^. However, the vast number of commensal symbiotic bacteria that reside in the mammalian gastrointestinal tract also share common molecular signatures with pathogens and some of these commensals are recognized by TLRs and trigger complex physiological effects on the host^26^. For example, the interaction of TLR5 with flagellated gut commensals is a determinant of obesity in animal models^27^. Microbial recognition of some of these commensal bacteria by host PRRs are required for the long-term establishment of microbial colonization in neonates^28^, to prevent bacterial translocation in mice and for the activation of host protective regulatory T cell responses^29,30^. Separately, excessive production of pro-inflammatory mediators following activation of TLRs by these bacteria can also lead to immunopathology. Multiple redundant mechanisms therefore exist to both positively and negatively regulate the activation of these PRR-initiated signaling pathways^31,32^.

Since MAMP-PRR interactions contribute to the establishment and regulation of commensal-host homeostasis, it is important to identify PRRs involved in recognition of commensal strains with purported immunomodulatory properties such as *L. salivarius* UCC118. To address this question we screened a panel of well characterised Gram-positive *L. salivarius* strains isolated from different environmental sources for their effects on macrophage cytokine and chemokine responses. In addition, we investigated the specific contribution of TLR2, a PRR widely involved in recognition of ligands produced by Gram-positive bacteria such as di-acyl lipopeptide FSL-1 (recognised by TLR2/6), in addition to Gram-negative tri-acyl lipopeptide Pam3-csk4 (recognised by TLR1/2) to these responses^33^. Surprisingly, we found that *L. salivarius* induced cytokine responses from macrophages were largely independent of TLR2 but were dependent on MyD88 signaling. In addition, macrophages co-cultured with *L. salivarius* upregulated the expression of *Clec4e*/Mincle, a C-type lectin receptor widely associated with recognition of glycolipids on *Mycobacterium tuberculosis* and *Candida albicans*^34^.

## Results

### *Lactobacillus salivarius* strains induce cytokine responses that are TLR2 independent but MyD88 dependent in macrophages

We investigated the role of TLR2 and MyD88 in the recognition and response by murine macrophages to the Gram-positive bacterium *L. salivarius*. Live cells of thirty-three *L. salivarius* strains from different environmental sources (Table 1), control probiotic and pathogenic bacteria and individual TLR ligands were screened using MSD multi-plex cytokine assays for their ability to stimulate WT, TLR2^−/−^ and MyD88^−/−^ BMDMs to produce a panel of cytokines (TNF-α, IL-6, IL-10, IL-12p70, IL-1β and IFN-γ) and the chemokine KC/GRO. TLR2 agonists (Pam3csk4 and FSL-1) did not trigger TNF-α responses in TLR2^−/−^ BMDMs, but TLR4 agonist (LPS) triggered cytokine response at equal magnitude in both WT and TLR2^−/−^ BMDMs (Supplementary Fig. S1), thus confirming the phenotype of the TLR2^−/−^ macrophages used in this screen.

**Table 1:** List of bacterial strains and ligands used in this study.

Bacteria/ligand	Strain/ligand details	Origin/supplier
TLR4 agonist	LPS B5 (*E. coli* B5)	Invivogen
TLR2/6 agonist	FSL-1	Invivogen
TLR1/2 agonist	Pam3csk4	Invivogen
NOD1 agonist	TriDAP	Invivogen
TLR2 agonist	Heat killed *Listeria monocytogenes*	Invivogen
Mincle + TLR2 agonist	Heat killed *Mycobacterium tuberculosis*	Invivogen
L. salivarius*	UCC118	Human ileal-cecal region
*L. salivarius*	UCC119	Chicken caecum
*L. salivarius*	AH4231	Human ileal-cecal region
*L. salivarius*	AH4331	Human ileal-cecal region
*L. salivarius*	AH43310	Human ileal-cecal region
*L. salivarius*	AH43324	Human ileal-cecal region
*L. salivarius*	AH43348	Human ileal-cecal region
*L. salivarius*	DSM20492	Human saliva
*L. salivarius*	DSM20554	Human saliva
*L. salivarius*	DSM20555	Human saliva
*L. salivarius*	NCIMB8816	Italian human saliva
*L. salivarius*	NCIMB8817	Turkey feces
*L. salivarius*	NCIMB8818	St. Ivel cheese
*L. salivarius*	NCIMB702343	Unknown
*L. salivarius*	CCUG27530B	Human abdomen, abscess
*L. salivarius*	CCUG38008	Human gall, 73-year-old
*L. salivarius*	CCUG43299	Human blood
*L. salivarius*	CCUG45725	Human blood
*L. salivarius*	CCUG47825	Human blood, 55-year-old
*L. salivarius*	CCUG44481	Bird
*L. salivarius*	CCUG47171	Human tooth plaque
*L. salivarius*	CCUG47826	Human blood, 55 year old
*L. salivarius*	JCM1040	Human intestine
*L. salivarius*	JCM1042	Human intestine
*L. salivarius*	JCM1044	Human intestine
*L. salivarius*	JCM 1045	Human intestine
*L. salivarius*	JCM1046	Swine intestine
*L. salivarius*	JCM1047	Swine intestine
*L. salivarius*	JCM1230	Chicken intestine
*L. salivarius*	01M14315	Human gall bladder pus
*L. salivarius*	LMG14476	Cat with myocarditis
*L. salivarius*	LMG14477	Parakeet with sepsis
B. breve*	UCC2003	NCIMB8807
E. coli*	EC101	Law et al.^a^
S. typhimurium*	SJW1103	Mirelles et al^b^
L. monocytogenes*	EDGe	NCTC7973
*L. rhamnosus*	GG	ATCC 53103

The table details Toll Like Receptor (TLR) ligands and their source of origin as well as bacteria species, strains and their source of origin, where known. *L. salivarius- *Lactobacillus salivarius*, *B. breve- Bifidobacterium breve*, *E. coli- Escherichia coli, L. rhamnosus*- *Lactobacillus rhamnosus*, *S. typhimurium*- *Salmonella typhimurium, L. monocytogenes- Listeria monocytogenes.*

^a^Law et al. 1995.

^b^Mireles et al. 2001.

**Figure 1:**
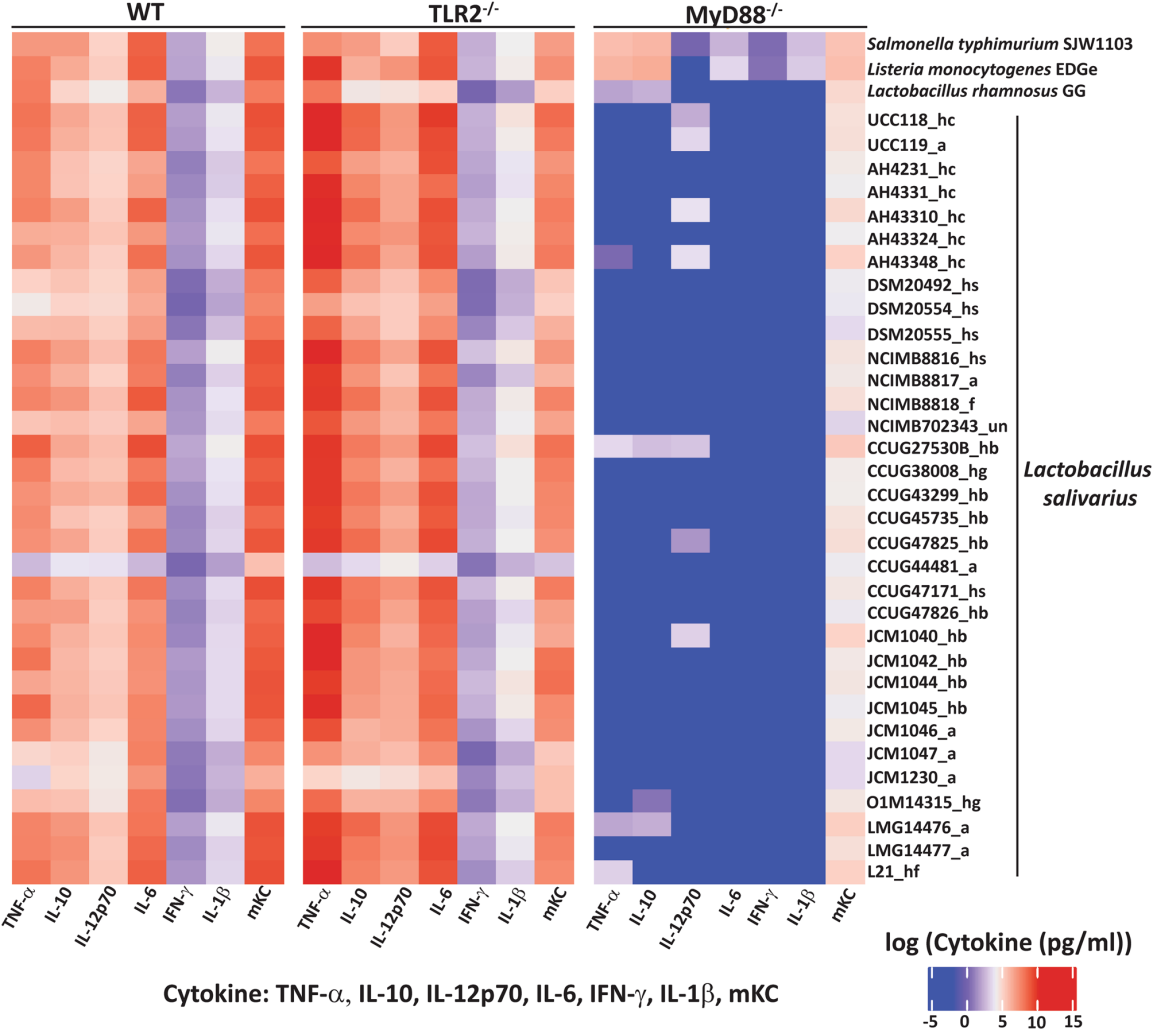
*L. salivarius* induced TLR2 independent but MyD88 dependent cytokine responses in mouse BMDMs in a strain independent manner. The heat map represents TNF-α, IL-10, IL-12p70, IL-6, IFN-ɣ, IL-1β and mKC responses in WT, MyD88^−/−^ and TLR2^−/−^ BMDMs treated with *Listeria monocytogenes* EDGe, *Lactobacillus rhamnosus* GG, *Salmonella typhimurium* SJW1103 and *L. salivarius* strains isolated from human ileal caecal region (hc), saliva (hs), blood (hb), gall bladder (hg) animals (a), food (f) or unknown sources (un). BMDMs were treated with the respective ligands or bacteria at MOI of 10 for 20 h followed by cytokine expression analysis using MSD 7-plex assay. The gradient of the heat maps generated represent log of median of cytokine concentration (pg/ml) and goes from blue to red (on a range of − 5 to + 15). Data shown are the average of 3 independent experiments (n = 3).

Irrespective of the environmental source of the *L. salivarius* strains, cytokine responses triggered by these bacterial strains were not reduced in TLR2^−/−^ BMDMs in comparison to WT BMDMs (Fig. 1). Indeed, we observed an increased cytokine response from TLR2^−/−^ BMDMs in these initial screens. The TLR2 independent cytokine responses triggered by *L. salivarius* did not correlate with the host species or tissue of origin of the strains (Table 1). The human commensal strain, *L. salivarius* UCC118 induced highest levels of cytokines, whereas *L. salivarius* CCUG44481 and the probiotic *L. rhamnosus* GG (LGG) induced the lowest, in WT and TLR2^−/−^ BMDMs (Fig. 1).

**Figure 2:**
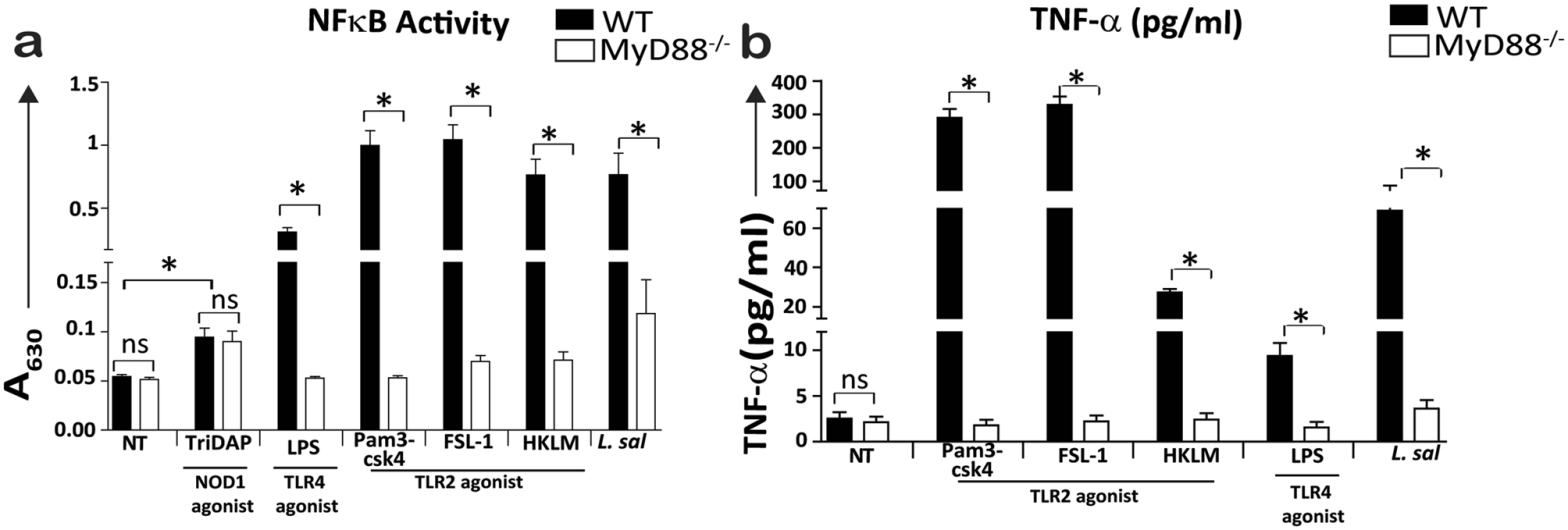
*L. salivarius* UCC118 stimulated NF-κB activity and cytokine responses were MyD88 dependent in THP-1 monocytes. (**a**) NF-κB transcriptional activity and (**b**) TNF-α cytokine secretion was measured in WT and MyD88^−/−^ THP1 cell lines that were non treated (NT) or treated with NOD1 agonist (TriDAP), TLR2 agonist (HKLM- heat-killed *Listeria monocytogenes*), TLR1/2 agonist (Pam3csk4), TLR2/6 agonist (FSL-1), TLR4 agonist (LPS) or co-cultured with *L. salivarius* UCC118 at MOI of 10 for 20 h. NF-κB transcriptional activity was measured indirectly by quantifying the activity of an NF-κB regulated reporter protein: secreted embryonic alkaline phosphatase, by using QUANTI-Blue (Invivogen) whose colour changes from blue to pink at 630 nm (A_630_) in THP1-XBlue and THP1-XBlue-defMyD cell lines upon addition of substrate to the cells. TNF-α cytokine secretion in THP1-XBlue and THP1-XBlue-defMyD cell lines were quantified by ELISA. Data shown are the average of triplicate wells of three independent experiments (n = 3). Statistical analysis was performed with two-tailed t-test in GraphPad Prism. p < 0.05 (denoted by *) is considered statistically significant. *ns* nonsignificant.

However, MyD88^−/−^ macrophages did not produce cytokines or chemokines in response to stimulation with *L. salivarius* strains, while exposure to two intracellular pathogens (Gram-negative *Salmonella typhimurium* SJW1103 and Gram-positive *Listeria monocytogenes* EDGe) resulted in production of TNF-α, IL-10 and KC/GRO, at fourfold lower levels compared to WT macrophages (Fig. 1). In addition, *L. salivarius* UCC118 was unable to induce NF-κB driven reporter activity (Fig. 2a) and TNF-α secretion (Fig. 2b) in MyD88^−/−^ THP-1 human monocyte-like cells thus confirming the requirement of MyD88 signaling for *L. salivarius* induced cytokine responses in macrophages. TriDAP (a NOD1 agonist), activated NF-κB driven reporter activity at comparable levels in WT and MyD88^−/−^ THP-1 cells thus confirming the capacity for MyD88-independent activation of NF-κB in these cell lines (Fig. 2a). Since TLR2 was dispensable for inducing cytokine responses by *L. salivarius* UCC118, we investigated if the ability to trigger TLR2-independent cytokine responses was a general feature of Gram-positive commensal bacteria by measuring cytokine responses triggered by *Bifidobacterium breve* UCC2003 (*B. breve*) in WT and TLR2^−/−^ BMDMs. *B. breve* is another sub-dominant member of the adult human gut microbiota and one of the first colonizers of the human gastrointestinal tract^35,36^ which has been shown to have immunomodulatory and other probiotic properties owing to the presence of an exopolysaccharide (EPS) on its cell surface^37^.

**Figure 3:**
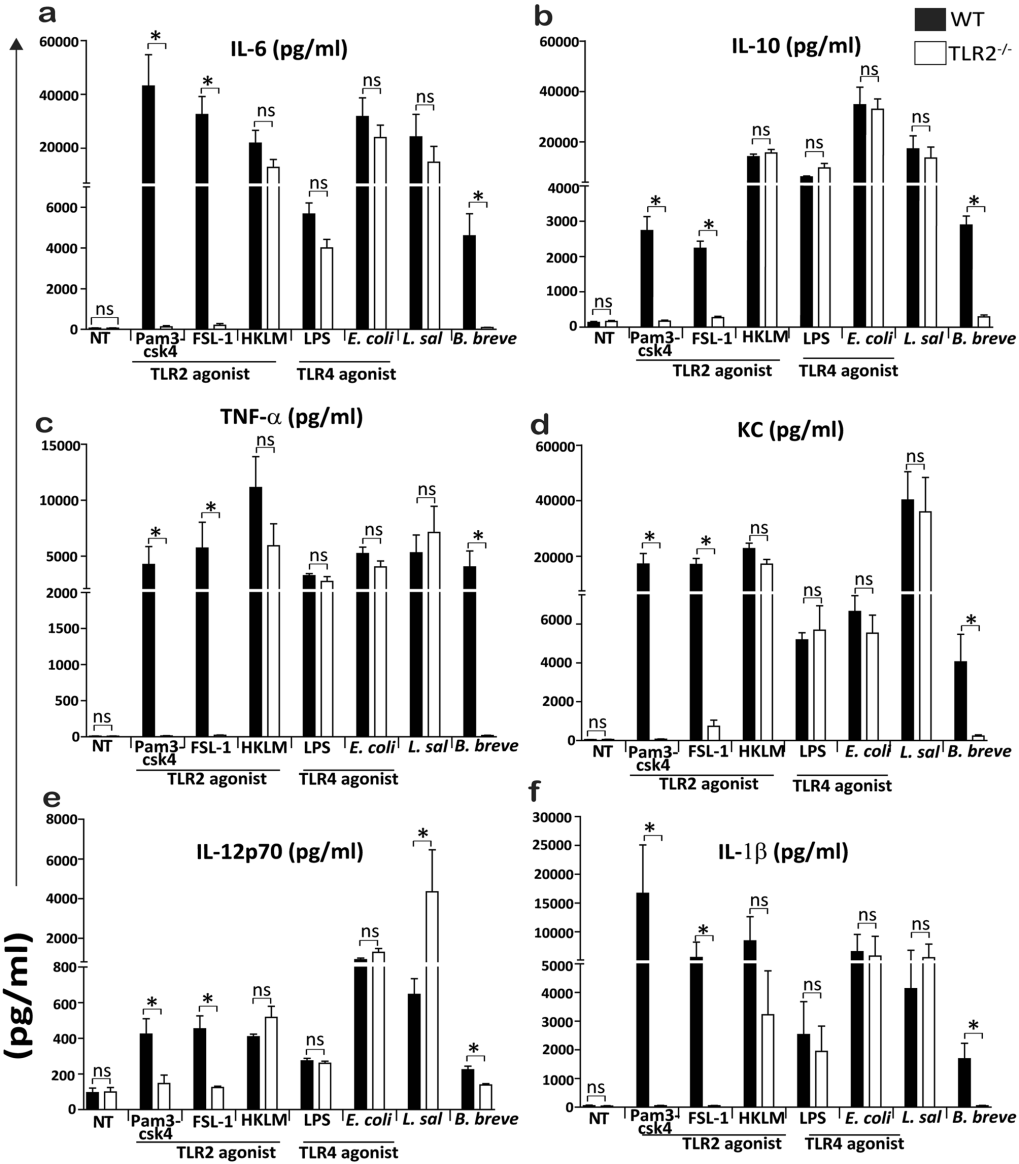
*L. salivarius* triggered cytokine responses are TLR2 independent but B. breve triggered cytokine responses are TLR2 dependent in BMDMs. (**a**) IL-6, (**b**) IL-10, (**c**) TNF-α, (**d**) KC, (**e**) IL-12p70 and (**f**) IL-1β cytokine secretion (pg/ml) was quantified by MSD-7 plex assay from WT and TLR2^−/−^ BMDMs that were non treated (NT) or treated with TLR2 agonist (HKLM- heat-killed *Listeria monocytogenes*), TLR1/2 agonist (Pam3csk4), TLR2/6 agonist (FSL-1), TLR4 agonist (LPS) or with *E. coli*, *L. salivarius* UCC118, and *B. breve* at MOI of 10 for 20 h. Data shown are the average of triplicate of three independent experiments (n = 3). Statistical analysis was performed with 2 tailed student t test in GraphPad Prism, p < 0.05 (denoted by *) was considered statistically significant. *ns* nonsignificant.

In contrast to the TLR2-independent nature of macrophage responses induced by *L. salivarius* UCC118, *B. breve* responses were completely dependent on TLR2 (Fig. 3a–f). Thus, *L. salivarius* UCC118 induced cytokine responses in macrophages are TLR2- independent but MyD88 dependent. Notably, *L. salivarius* UCC118 induced IL-12p70 responses were ~ eightfold higher in TLR2^−/−^ macrophages compared to the response seen in WT macrophages (Fig. 3e). TLR2 agonists (Pam3csk4 and FSL-1) were used as positive controls and TLR4 agonists (LPS and *E. coli*) were used as negative controls to confirm the TLR2^−/−^ phenotype of these macrophages. Cytokine responses to LPS and *E. coli* (TLR4 agonists) were TLR2-independent, cytokine responses to Pam3csk4 and FSL-1 (TLR2 agonists) were TLR2-dependent but cytokine responses to HKLM (TLR2 agonist) was TLR2*-*independent (Fig. 3a–f)^38^.

### *L. salivarius* UCC118 cytokine responses are TLR4 independent in murine BMDMs

**Figure 4:**
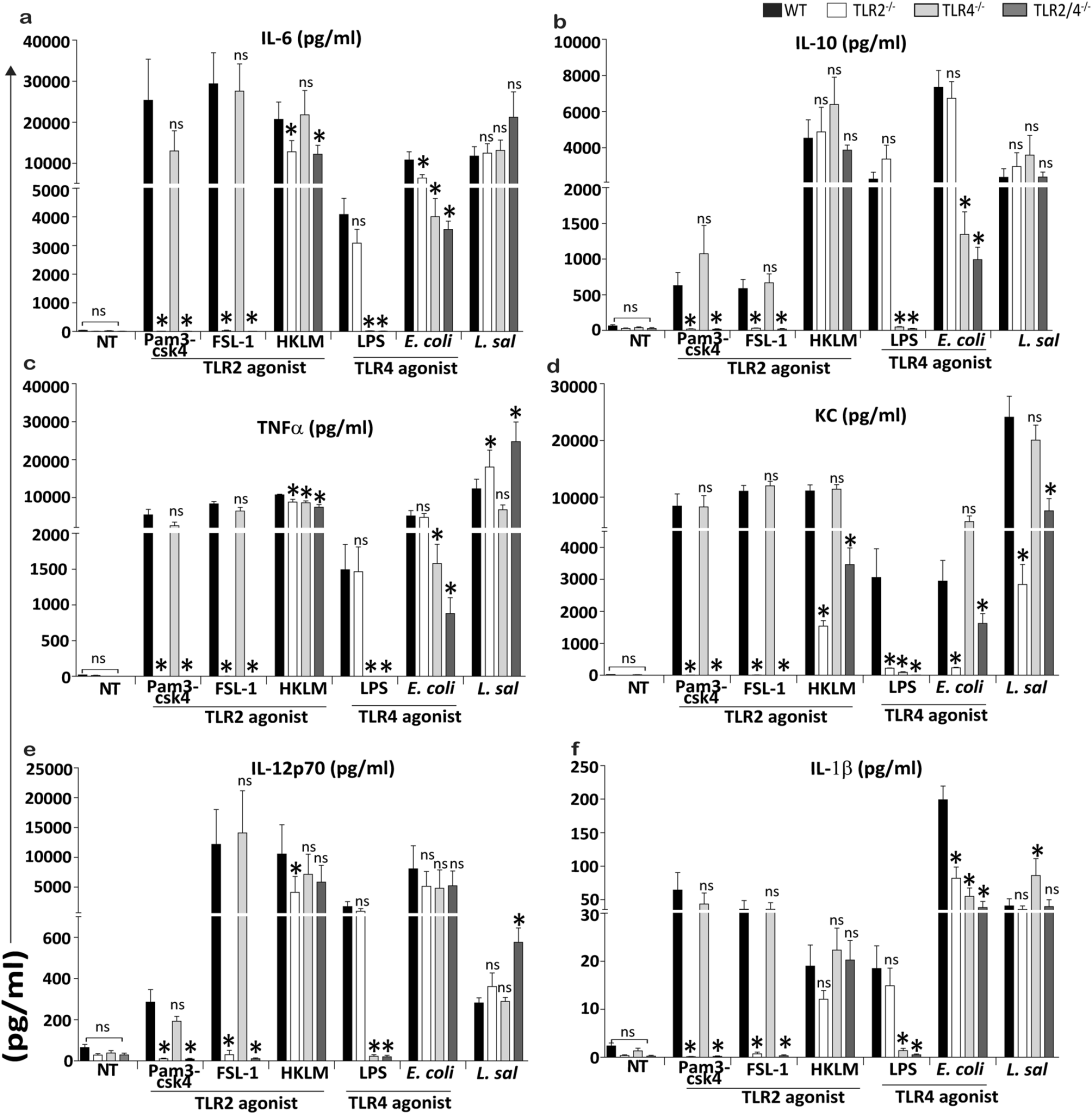
*L. salivarius* induced cytokine responses are TLR4 independent in murine BMDMs. (**a**) Il-6, (**b**) Il-10, (**c**) TNF-α, (**d**) KC, (**e**) Il-12p70 and (**f**) Il1-β cytokine secretion (pg/ml) was quantified by MSD-7 plex from WT and TLR2^−/−^, TLR4^−/−^, TLR2/4^−/−^ BMDMs that were non treated (NT) or treated with TLR2 agonist (HKLM- heat-killed *Listeria monocytogenes*), TLR1/2 agonist (Pam3csk4), TLR2/6 agonist (FSL-1), TLR4 agonist (LPS- B5 from *E. coli* B5) or co-cultured with *E. coli* and *L. salivarius* UCC118 at MOI of 10 for 20 h. Data shown are the average of triplicate of three independent experiments (n = 3). Cytokine response triggered by TLR2^−/−^, TLR4^−/−^ and TLR2/4^−/−^ BMDMs were compared relative to the cytokine response triggered by WT BMDMs. Statistical analysis was performed with 2 tailed student t test in GraphPad Prism, p < 0.05 (denoted by *) was considered statistically significant. *ns* nonsignificant.

**Figure 5:**
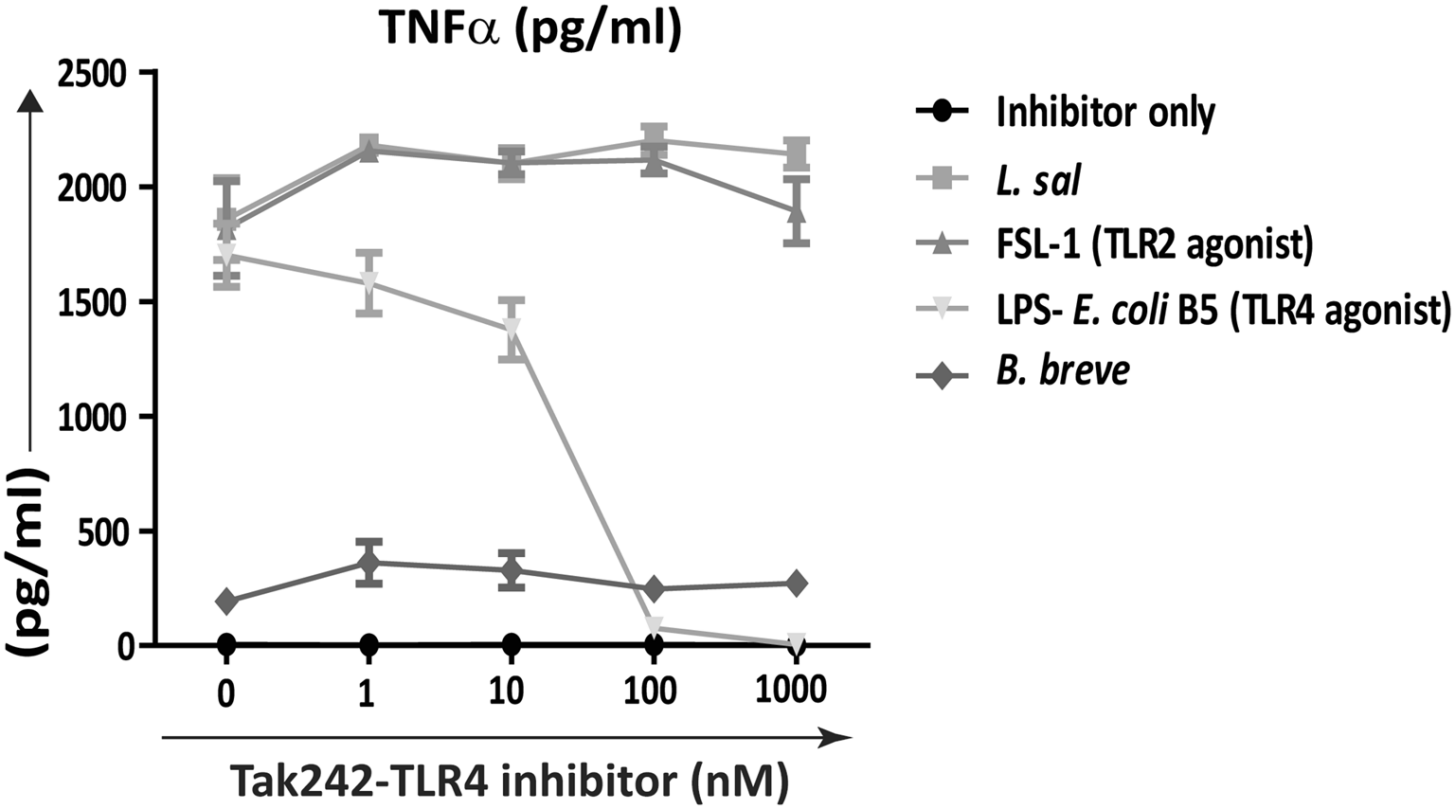
*L. salivarius* induced cytokine responses are TLR4 independent in Tak242 treated murine WT BMDMs. WT BMDMs were either treated with TLR4 inhibitor (Tak242) alone (inhibitor only) at the indicated concentrations or Tak242 was co-treated with TLR2/6 agonist (FSL-1), TLR4 agonist (LPS) or co- cultured with *B. breve* and *L. salivarius* UCC118 at MOI 10 for 20 h. TNF-α concentration (pg/ml) was analysed post 20 h using TNF-α ELISA duosets. Data shown are the average of triplicate wells of three independent experiments (n = 3).

TLR4 is a PRR required for recognition of the Gram-negative bacterial cell-envelope component, LPS. However, recent studies suggest that TLR4 also contributes to the recognition of Gram-positive bacteria such as *Streptococcus pneumoniae*^39^*.* It is also required for triggering protective host responses in response to exopolysaccharide (EPS) from Gram-positive *Lactobacillus spp.* and *Bacillus spp.* and from Gram-negative bacteria like *Bordetella spp.*^40–42^. Since EPS production is an important characteristic of *Lactobacillus spp.*^43,44^, we assessed if TLR4 was required for recognition of *L. salivarius* UCC118 and *L. salivarius* UCC118 mediated cytokine responses in BMDMs. Neither TLR2^−/−^, TLR4^−/−^ nor TLR2/4^−/−^ BMDMs elicited differential cytokine responses compared to WT BMDMs after co-culture with *L. salivarius* UCC118 (Fig. 4a–f). Blocking TLR4 receptor activation in WT BMDMs using the TLR4 selective inhibitor Tak242^38^ prevented induction of TNF-α responses by LPS but not by *L. salivarius* UCC118 (Fig. 5). This suggested that *L. salivarius* UCC118 induced cytokine responses in BMDMs were TLR2 and TLR4 independent.

**Figure 6:**
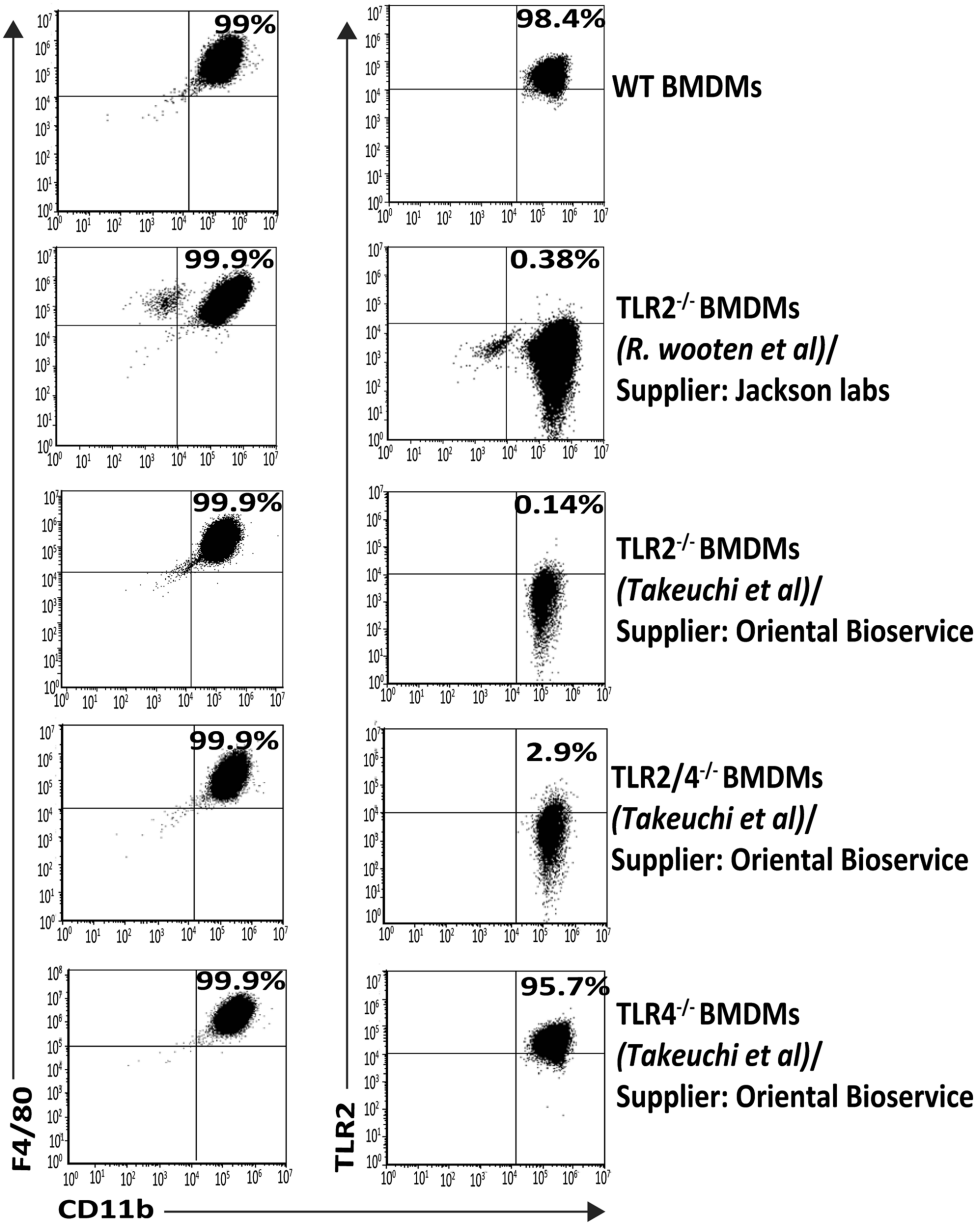
Characterisation and validation of in vitro generated mouse BMDMs. BMDMs were stained with CD11b-PECy7, F4/80-FITC and TLR2-PE and analysed by flow-cytometry. CD11b vs F4/80 and CD11b vs TLR2 dot plots were obtained by gating on cells with CD45 expression. Numbers in the quadrants indicate the percentage of CD11b^+^F4/80^+^ cells and the CD11b^+^TLR2^+^ cell in the respective quadrant. BMDMs were generated from WT mice, TLR2^−/−^ mice purchased from Oriental Bioservice (generated by Osam u Takeuchi et al^33^) and TLR2^−/−^ mice from Jackson laboratories (generated by R. Mark Wooten et al^39^) as indicated.

Because of the surprising TLR2-independent nature of the cytokine responses to *L. salivarius* UCC118 from BMDMs derived from TLR2^−/−^ mice purchased from Jackson labs^39^ (Fig. 3a–f), we also compared cytokine responses to *L. salivarius* UCC118 from BMDMs generated from different TLR2^−/−^ mice purchased from Oriental Bioservice^33^ **(**Fig. 4a–f**)** in order to exclude confounding variables such as effects from different genetic backgrounds, vendors or methods used to generate the targeted KOs (knockout). Cytokine responses to LPS and *E. coli* (TLR4 agonists) were TLR2-independent, but TLR4-dependent (Fig. 4a–f). Cytokine responses to Pam3csk4, FSL-1 were TLR2-dependent as observed in mice from Jackson labs (Fig. 3) but cytokine responses to HKLM (TLR2 agonist) were TLR2-dependent in BMDMs from mice purchased from Oriental Bioservice (Fig. 4a–f) as opposed to BMDMs from mice sourced from Jackson labs (Fig. 3a–f). Interestingly, in BMDMs generated from mice sourced from Oriental Bioservice, increased production of KC in response to *L. salivarius* UCC118 was abrogated in TLR2^−/−^ and TLR2/4^−/−^ compared to WT BMDMs (Fig. 4d) while this effect was not observed in the BMDMs generated from TLR2^−/−^ mice purchased from Jackson labs (Fig. 3d). Another major difference was the increased Il12-p70 response from TLR2^−/−^ BMDMs generated from mice purchased from Jackson labs (Fig. 3e) and the increased TNF-α response (Fig. 4c) in mice purchased from Oriental Bioservice. All these BMDMs expressed classic macrophage markers, CD11b and F4/80 at identical levels as assessed by FACS, while as expected all TLR2^−/−^ BMDMs did not express TLR2 (Fig. 6). Thus, the TLR2-independent nature of the BMDM cytokine response to *L. salivarius* UCC118 was confirmed in BMDMs from two different strains of TLR2^−/−^ mice. However, we did observe different TLR2-independent responses which is suggestive of possible genetic differences between the different KO mice strains on the C57BL/6 background.

### *L. salivarius* UCC118 induced TNF-α response is partially Mincle receptor dependent

**Figure 7:**
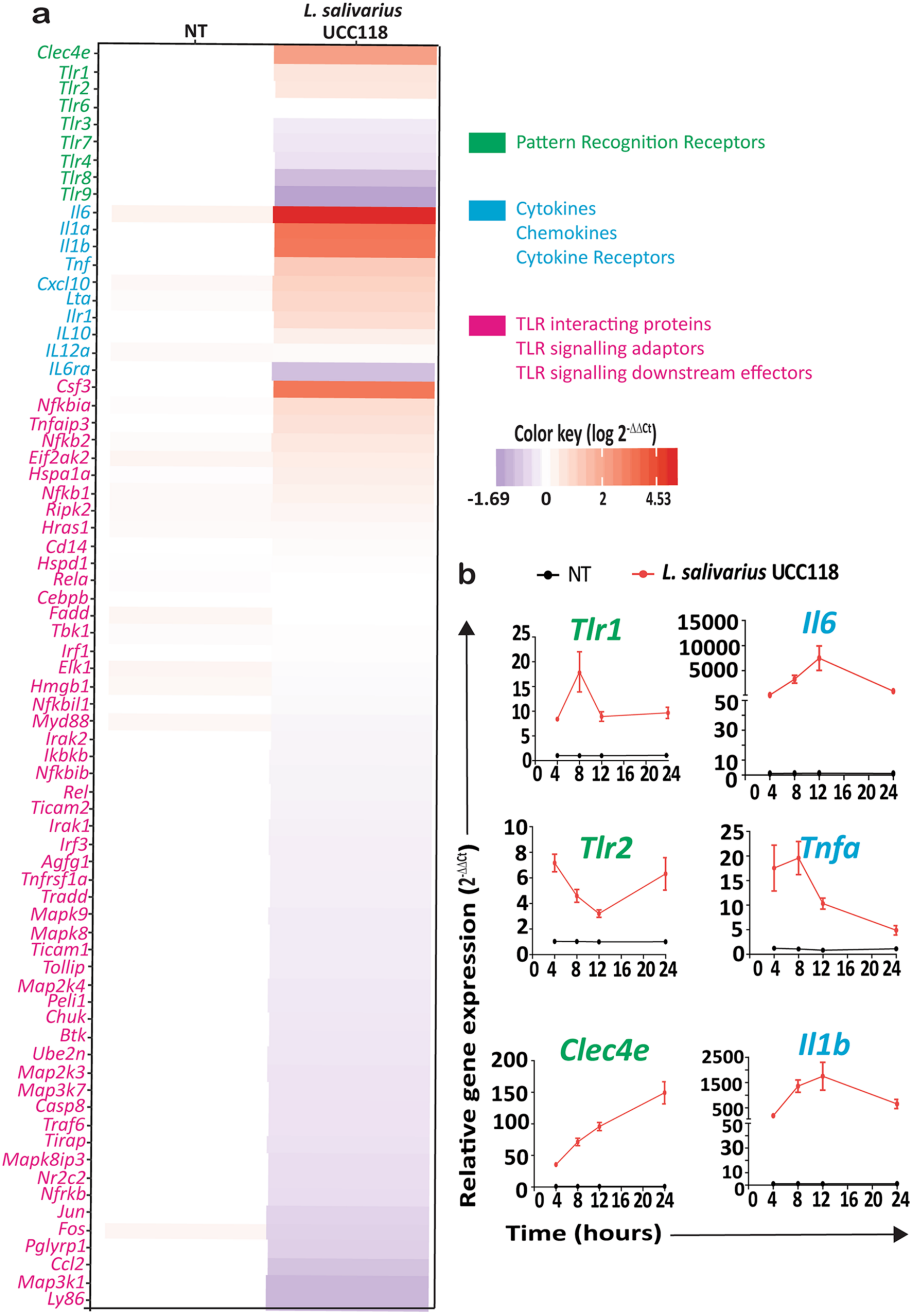
Clec4e is the most upregulated PRR in *L. salivarius* UCC118 co-cultured BMDMs. (**a**) Heat map represents log values of the relative fold difference in gene expression (log 2^−ΔΔCt^) of genes encoding pattern recognition receptors (green), cytokines and chemokine and cytokine receptors (blue), TLR interacting protein, signaling adaptors and downstream effectors (pink). WT BMDMs were either non-treated (NT) or cocultured with *L. salivarius* UCC118 at MOI of 10 for 20 h. (**b**) Relative fold expression of PRRs *Tlr1*, *Tlr2* and *Clec4e* and cytokines *Il6*, *Tnfa* and *Il1b* at 4, 8, 12 and 24 h in BMDMs that were either non-treated (NT) or cocultured with *L. salivarius* UCC118. Relative gene expression data of genes in both (**a**) and (**b**) are the average of three independent experiments (n = 3) normalised to relative expression of housekeeping gene *Actb* (beta- actin).

**Figure 8:**
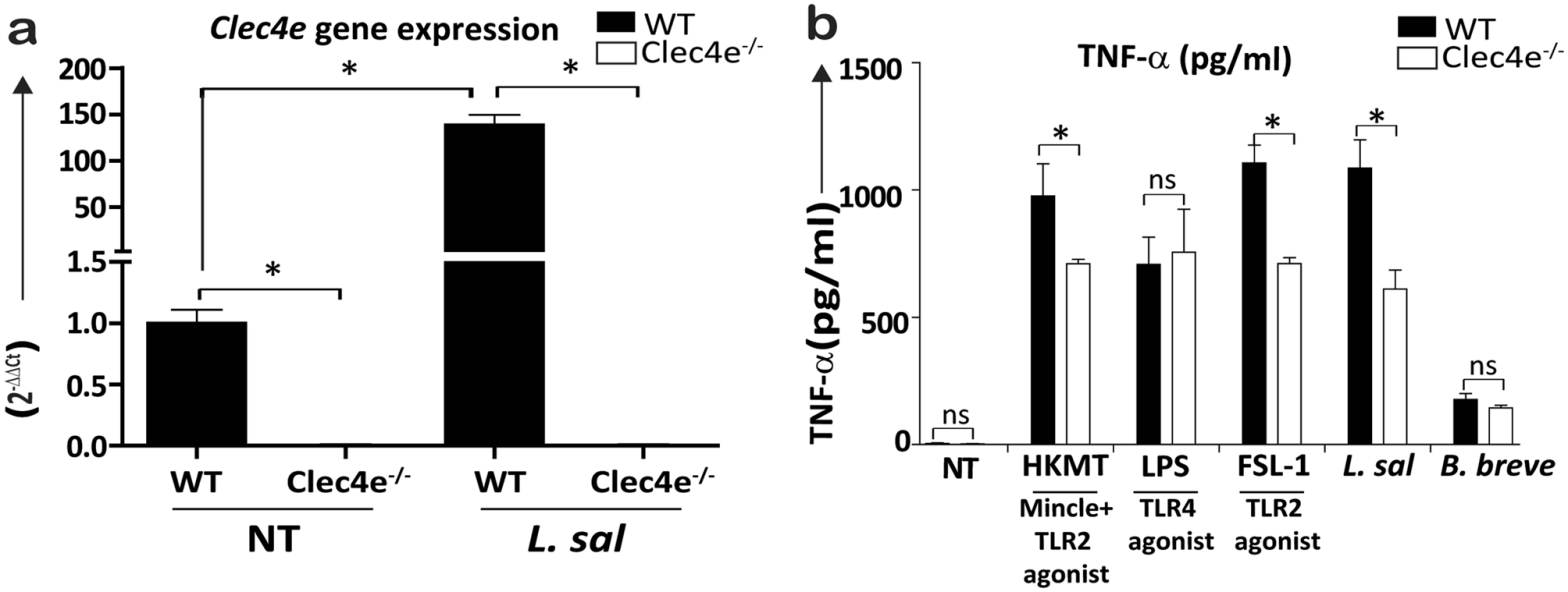
TNF-α response triggered by *L. salivarius* UCC118 and TLR2 agonist (FSL-1) is partially dependent on Clec4e (Mincle) receptor expression in BMDMs. (**a**) Relative gene expression of *Clec4e* was analysed in total RNA harvested from WT and Clec4e^−/−^ BMDMs that were either left untreated (WT-NT and Clec4e^−/–^NT) or treated with *L. salivarius* UCC118 (WT-UCC118 and Clec4e^−/–^UCC118) at MOI of 10 for 20 h co-culture. Data are the average of three independent experiments (n = 3) normalised to relative expression of the gene *Actb* (beta-actin). (**b**) TNF-α secretion (pg/ml) was measured in WT and Clec4e^−/−^ BMDMs that were either non treated (NT) or treated with TLR2/6 agonist (FSL1), TLR4 agonist (LPS), Mincle agonist (HKMT- heat killed *Mycobacterium tuberculosis*) or co-cultured with *L. salivarius* UCC118 and *B. breve* at MOI of 10 for 20 h. TNF-α secretion was quantified by mouse TNF-α ELISA duosets. Data are the average of three independent experiments (n = 3). Statistical analysis for both (**a**) and (**b**) was performed with 2 tailed student t test in GraphPad Prism, p < 0.05 (denoted by *) was considered statistically significant. *ns* nonsignificant.

Since neither TLR2 nor TLR4 were required for *L. salivarius* UCC118 induced cytokine responses, we analysed the expression of 75 PRR pathway-associated genes in WT BMDMs co-cultured with *L. salivarius* UCC118 for 20 h, in an attempt to identify PRRs involved in the recognition of *L. salivarius* UCC118. Most of the cytokine and chemokine genes (*Il6*, *Il1a*, *Il1b*, *Tnfa*, *Cxcl10*, *Csf3*) were significantly upregulated in BMDMs co-cultured with *L. salivarius* UCC118 in comparison to NT (non-treated) BMDMs (Fig. 7a). Among the PRR genes whose expression was upregulated, *Clec4e* coding for Mincle showed the greatest increased fold change, followed in rank order by the PRRs- *Tlr1* and *Tlr2* in *L. salivarius* UCC118 co-cultured with BMDMs. Expression of all the other TLRs (*Tlr3*, *Tlr4*, *Tlr7*, *Tlr8* and *Tlr9*) were downregulated while *Tlr6* expression did not vary between the groups in these BMDMs (Fig. 7a). Upregulation of *Tlr1*, *Tlr2*, *Clec4e*, *Il6*, *Tnfa* and *Il1b* was confirmed separately at 4, 8, 12 and 20 h by separate RT-qPCR experiments (Fig. 7b). Because *Clec4e* was the most upregulated PRR in BMDMs co-cultured with *L. salivarius* UCC118 we then investigated *L. salivarius* UCC118 induced cytokine responses in BMDMs from WT and Clec4e^−/−^ mice. Absence of *Clec4e* expression in Clec4e^−/−^ BMDMs was confirmed by RT-qPCR (Fig. 8a). Heat-killed *Mycobacterium tuberculosis* (HKMT), a reported Mincle and TLR2 agonist^45,46^, was used a positive control for TNF-α response triggered by *Clec4e*/Mincle in BMDMs. TNF-α response to HKMT was reduced in *Clec4e*^−/−^ macrophages as expected (Fig. 8b). TNF-α responses triggered by *L. salivarius* UCC118 were also partially reduced in *Clec4e*^−/−^ BMDMs in comparison to BMDMs from co-housed WT littermates (Fig. 8b). LPS (TLR4 agonist) and *B. breve* showed no difference in TNF-α responses between WT and *Clec4e*^−*/*−^ BMDMs (Fig. 8b). Interestingly, FSL1 (TLR2 agonist) also had reduced TNF-α responses in Clec4e^−/−^ BMDMs (Fig. 8b), suggesting a possible role for Mincle in the regulation of TLR2 induced cytokine responses. To identify if Mincle (*Clec4e*) is functionally associated with TLR2 via direct/indirect protein–protein interactions (PPI) in mice or humans, we used “Search Tool for Retrieval of Interacting Genes/Proteins” (STRING), an interactome database widely used to predict PPI^47^. STRING PPI network revealed that TLR2 and Mincle are co-expressed in both mouse and humans (Supplementary Fig. S2) supporting our observation of upregulated expression of *Tlr2* and *Clec4e* in *L. salivarius* UCC118 co-cultured BMDMs (Fig. 7). However, there was no evidence for a direct functional association between Mincle and TLR2 in either humans or mice, but putative homologs of Mincle (like CLEC18A, CLEC4M, CLEC7A) were found to interact with TLR2 in humans (Supplementary Fig. S2). Overall, these results suggested that Clec4e or its downstream sigalling components may be potentially important contributors to TLR2 mediated induction of cytokine responses by TLR2 agonist ligands (like FSL-1 and HKLM) and to responses to *L. salivarius* UCC118.

## Discussion

In this study we investigated the mechanisms underpinning the recognition of Gram-positive commensal bacterial species *Lactobacillus salivarius* by mouse macrophages and a human monocyte-like cell line. We found that macrophage cytokine responses to these bacteria were TLR2 independent yet completely MyD88 dependent and associated with the upregulation of *Tlr1, Tlr2* and *Clec4e* PRR genes in these cells. Since MyD88 signalling was required for triggering of macrophage cytokine responses to *L. salivarius,* it is possible that other MyD88-dependent pathways such as TLR7 and TLR9 and/or the IL-1R signalling pathway might be important contributors to *L. salivarius* recognition and response by these cells^48^. In fact, it is known that other commensal *Lactobacillus* species such as *L. reuteri* can activate IFN signalling through TLR7 in plasmacytoid dendritic cells^49^ and *L. plantarum* can induce elafin secretion via TLR9 in CaCO2 cells^50^. The involvement of MyD88 independent PRRs such as TLR3^51^ or NLRs (NOD like receptors)^52^ as the predominant players in transducing cytokine responses by these bacteria can be excluded given that *L. salivarius* trigger macrophage cytokine responses in a MyD88 dependent manner. In support of these observations, we found that while the stimulation of BMDMs with *L. salivarius* UCC118 downregulated the expression of *Tlr3, Tlr4, Tlr7, Tlr8* and *Tlr9,* it upregulated *Tlr1, Tlr2 and Ilr1* which signal in a MyD88 dependent manner. Interleukin-1 receptor (IL-1R) mediated signaling might be another contributing factor involved in *L. salivarius* induced cytokine responses. *L. salivarius* induced the expression of *Il1a, Il1b* and *Ilr1* genes (Fig. 7) and secretion of IL1β cytokine when they were co-cultured with bone-marrow derived macrophages. MyD88 acts as an adaptor component in the IL-1R signaling pathway^53–55^. Thus, it is possible that IL-1α or/and IL1β secreted upon initial recognition and internalization of *L. salivarius* by macrophages might participate in the further induction of cytokine responses in an autocrine and paracrine manner by signalling through IL-1R in a Myd88 dependent manner. This would explain the reduced cytokine response observed in Myd88^−/−^ macrophages. In addition, stimulation of BMDMs with *L. salivarius* UCC118 was also able to induce an unexpected hyper-inflammatory cytokine response in TLR2^−/−^ BMDMs. This suggests a possible immunoregulatory role of TLR2 in modulating macrophage responses to *L. salivarius*. It is possible that in addition to its importance in host cells for the initiation of a protective inflammatory response against invading pathogens TLR2 might also have evolved an alternative immunoregulatory role or other protective function in the context of host-commensal interactions^56^. Indeed, bacterial components of the microbiota are able to directly downregulate some downstream MyD88 effector genes in zebra fish at steady-state through TLR2^57^. An immunoregulatory role for TLR2 has also been previously suggested in disease contexts such as in mouse models of arthritis^58^ and colitis^59,60^. Although addressing the mechanistic basis and significance of this observed phenotype in TLR2^−/−^ BMDMs is beyond the scope of this study, this aspect should be further explored in the future.

The purity of BMDMs and TLR2 status were validated by flow cytometry. The TLR2 function of BMDMs used in this study were validated using selective TLR2 agonists-Pam3csk4, FSL1 and HKLM. We observed some notable differences in cytokine responses to stimulation with whole bacteria versus individual MAMPs between BMDMs generated from TLR2 KO mice sourced from two different suppliers used in this study. These included, a diminished cytokine response against HKLM and abrogated KC responses against all treatments in TLR2^−/−^ BMDMs derived from the mice purchased from Oriental Bioservices, which could not be observed in BMDMs from the mice purchased from Jackson Labs. Nonetheless, the TLR2-dependent nature of the responses to individual TLR2-selective MAMPs, and to the commensal bacteria *B. breve* and the TLR2-independent nature of cytokine responses to *L. salivarius* UCC118 was consistently observed in BMDMs generated from both TLR2^−/−^ mouse strains sourced from these suppliers. This highlights the importance of carrying out similar in vitro and in vivo experiments in mice of different genetic backgrounds, breeding facilities, suppliers and/or vendors before making major conclusions of a particular phenotype as the observed effects may be affected by these confounding factors^61,62^.

The host has evolved multiple primary and secondary protective barrier-associated mechanisms to contain commensal bacteria within the gut lumen. Despite this, exposure of host innate immune cells to members of the microbiota through various combinations of MAMP-PRR interactions might still occur in the gut lamina propria or in systemic compartments in the context of pathologies such as inflammatory bowel disease (IBD). In this context, certain members of the gut microbiota may take advantage of the compromised gut barrier to translocate from the intestinal tract into the systemic circulation^63^. Defective TLR1 signaling by intestinal epithelial cells has been associated with disruption of intestinal homeostasis and increased inflammatory responses against microbiota^64^ and defective MyD88 signaling is linked to reduced antibacterial responses and enhanced adherence and translocation of gut-associated bacteria from lumen to epithelial cells^29,65^. In this study we chose a widely studied gut-associated commensal bacteria: *L. salivarius* in order to understand the relative contribution of TLR2 to their recognition and subsequent triggering of NF-κB dependent cytokine responses in macrophages. The expression of *Clec4e*, *Tlr1* and *Tlr2* were significantly upregulated in BMDMs co-cultured with *L. salivarius* UCC118. Clec4e (also known as Mincle) is a C-type lectin receptor that is broadly known to recognize mannosyl fatty acids ligands associated with *Mycobacterium tuberculosis* bacteria and *Candida albicans* fungus and triggers intracellular signaling through its adaptor Syk (spleen tyrosine kinase)^34,66,67^. Clec4e PPI network retrieved from STRING database suggested co-expression of Clec4e and TLR2 as well as functional association between Clec4e homologs and TLR2 in both mice and humans. However, a recent study has reported direct interaction of Clec4e itself with TLR2^68^. Lipomannan and Lipoarabinomannan from Corynebacterium have been shown to upregulate cell surface Clec4e expression in macrophages through a TLR2- MyD88 pathway and Clec4e consequentially binds to Corynebacterium glycolipids to induce NF-кB activation and induction of inflammatory responses including nitrite and granulocyte colony stimulating factor (G-CSF) secretion thus supporting a co-operation between TLR2 and Clec4e to sense bacterial glycolipids^68^. Glycolipids derived from *L. plantarum* are also able to trigger transcription factor nuclear factor of activated T-cells (NFAT) in reporter cell lines through human and mouse mincle^69^. A recent study also shows that microbial interaction with Clec4e is important in triggering cytokine production by DCs (dendritic cells), for the promotion of intestinal barrier integrity and prevention of translocation of gut bacteria to systemic tissues at steady-state^70^. In addition, *Lactobacillus sps.* were recognised as having the highest binding affinity to Clec4e amongst other commensal bacteria reported in this study^70^. Our study also supports the possibility of *Lactobacillus* derived ligands binding to Clec4e since macrophage TNF-α responses to *L. salivarius* UCC118 were partially dependent on Clec4e. Clec4e has been recently reported to contribute to phagocytic function and to control intracellular growth of *Mycobacterium tuberculosis* by triggering autophagy^71^. Clec4e expression increased steadily from 4 to 24 h in BMDMs co-cultured with *L. salivarius*. Thus, it is also possible that Clec4e may act as phagocytic receptor to trigger internalization of *L. salivarius* by macrophages. In the future, it would be of interest to identify if there are glycolipids in *L. salivarius* similar to that in *L. plantarum* which could mediate cytokine responses directly through Clec4e and to investigate if Clec4e might have a role in the internalization of *L. salivarius* in addition to its importance in the induction of cytokine responses.

Our study also provides evidence of the differential requirements for individual PRR pathways in macrophage cytokine responses to Gram-positive commensal bacteria since *B. breve* required TLR2 to induce cytokine responses in macrophages but *L. salivarius UCC118* did not even though both of these bacteria were Gram-positive. The difference in the induction of macrophage cytokine responses by these two bacteria maybe attributed to differences in *L. salivarius* cell surface architecture^72,73^. Collectively, our data supports a model whereby the response of host macrophages to *L. salivarius* is TLR2 and TLR4 independent but MyD88-dependent. Our data also suggests Mincle (*Clec4e*) as an important contributor to TLR2-dependent responses against TLR2 ligands as well as to TLR2-independent responses to bacteria like *L. salivarius*. These observations support a model whereby the integration of signals from different PRR pathways and MyD88-dependent pathways may ultimately determine immune responses to commensal bacteria at the host-microbe interface.

## Materials and methods

### Mice

All mice used in this study were 8–12 week old male mice from a C57BL/6 background. TLR2^−/−^, TLR4^−/−^, TLR2/4^−/−^ mice and WT (wildtype) controls were obtained from Oriental BioService (Kyoto, Japan) and TLR2^−/−^, MyD88^−/−^ and WT controls were purchased from Jackson Laboratories (Bar Harbor, USA). All these animals were housed in the Biological Service Unit animal housing facility at University College Cork (UCC) under specific pathogen-free (SPF) conditions using individually ventilated cages (IVC). Standard housing and environmental conditions were maintained (temperature 21 °C, 12 h light and 12 h darkness with 50% humidity). Animals were fed regular chow food (purchased from Envigo (Cambridgeshire, UK). Animals were also given water ad libitum. WT and Clec4e^−/−^ mice were littermates born from heterozygous parents and housed in the animal housing facility at Centro Nacional de Investigaciones Cardiovasculares Carlos III (CNIC) until taken for bone-marrow isolation. Mice at CNIC were housed under SPF conditions using IVC. Standard housing and environmental conditions were maintained (temperature 21 °C, 12 h light and 12 h darkness with 50% humidity). Animals were fed with chow diet (LASQC diet, Altromin international, Lage, Germany). Animals were also given water ad libitum. For in vitro analysis of cytokine responses bone-marrow derived macrophages (BMDMs) were generated from WT, TLR2^−/−^, TLR4^−/−^, TLR2/4^−/−^ and Clec4e^−/−^ mice (n = 3, age: 8–12 weeks, all males), and experiments were repeated a total of three times (n = 3) in technical triplicates per experiment. For gene expression analysis, RNA was harvested from WT or Clec4e^−/−^ (n = 3) BMDMs that were either nontreated (NT) or *L. salivarius* UCC118 co-cultured and experiments were repeated a total of three times (n = 3) with technical duplicates per experiment. All animal work in CNIC was approved by the local animal ethics committee. All animal procedures in CNIC conformed to EU Directive 2010/63EU and Recommendation 2007/526/EC regarding the protection of animals used for experimental and other scientific purposes, enforced in Spanish law under Real Decreto 1201/2005. All animal work in UCC was approved by the Animal Experimentation Ethics Committee (AEEC) of University College Cork, License B100/4104 and Euthanasia Only—Application ID 2018/009. All animal work and procedures in UCC were performed in accordance with EU legislation, in accordance with EU Directives 86/609/EEC and 2010/63/EU, for the protection of animals used for scientific purposes.

### Bacterial strains and culture conditions

*Lactobacillus salivarius* and other bacterial strains used in this study and their sources are listed in Table 1. *Lactobacillus* strains were routinely cultured at 37 °C under micro-aerobic conditions (5% CO2) in de Man Rogosa-Sharpe medium (MRS, Difco). *Bifidobacterium breve* UCC2003 was cultured in reinforced clostridium medium (RCM, Sigma-Aldrich) for 10 h, and subcultured in MRS supplemented with 1% cysteine overnight at 37 °C in anaerobic conditions. *E. coli* EC101 was grown aerobically in Luria–Bertani broth (LB, Sigma-Aldrich) at 37 °C and 200 rpm. Multiplicity of infection (MOI) values of these bacteria for co-culture experiments were calculated by measuring their cell numbers corresponding to their optical density values at 600 nm (OD_600_) after overnight (16 h) incubation. OD 1 of 16 h culture of *L. salivarius* UCC118 corresponded to 2 × 10^8^ cells/ml while OD 1 of overnight culture of *B. breve* UCC2003 and *E. coli* EC101 corresponded to 10^9^ cells/ml.

### Cell lines and culture conditions

THP1-XBlue cells (thpx-sp) and THP1-XBlue-defMyD cells (thpx-dmyd) were purchased from Invivogen and routinely cultured in Roswell Park Memorial Institute medium (RPMI 1640, Sigma) supplemented with 10% foetal calf serum (Sigma-Aldrich) at 37֩c in 5% CO_2_. BMDMs were prepared as described elsewhere^74^ with the following modification, 7 days post differentiation cells were dissociated using StemproAccutase (Thermofisher Scientific).

### Cytokine bioassay

MAMP ligand treatments and bacterial co-culture experiments were carried out on THP1 cell lines or BMDMs, in 100 µl total volume of RPMI or Dulbeccos Minimal Essential Media (DMEM, Sigma) supplemented with 10% FCS respectively. List of ligands used in this study and their targets are listed in Table 1. Macrophage cells were seeded at 50,000 cells per well in flat bottom 96 well plates and co-cultured or stimulated for 20 h with (a) *L. salivarius* strains (b) *B. breve* UCC2003 (c) *E. coli* EC101 at a MOI of 10 bacteria per macrophage and (d) HKLM (heat killed *Listeria monocytogenes)* (tlrl-hklm, Invivogen)—10^8^ cells/ml, (e) LPS from *E. coli* B5 (tlrl-b5lps, Invivogen)- 100 ng/ml, (g) Pam3csk4 (tlrl-pms, Invivogen)- 300 ng/ml, (h) FSL-1—(tlrl-fsl, Invivogen) 100 ng/ml (i) TriDAP (tlrl-tdap, Invivogen)- 100 ng/ml (j) HKMT (heat killed *Mycobacterium tuberculosis*) (tlrl-hkmt-5, Invivogen)—100 µg/ml. HKMT is a Mincle and TLR2 agonist. Invivogen recommend the following working concentrations; 10–100 µg/ml when used as a Mincle ligand or 100 ng–10 µg/ml when used as a TLR2 ligand. Cells were incubated at 37 °C in 5% CO_2_ at specified concentrations for 20 h after which supernatants were collected for analysis of secreted TNF-α, IL-10, IL-12p70, IL-1β, IFN-γ and mKC using 7-plex MSD assays (MesoScale Discovery Gaithersburg, MD) or ELISA Duosets for TNF-α (DY410-05, R&D Biosystems) and IL-10 (DY417-05, R&D Biosystems) in accordance with the manufacturer’s instructions. NF-κB transcriptional activity in THP1 reporter cell lines was assayed using Quanti-blue (rep-qb1, Invivogen), in accordance with the manufacturers instructions.

### Quantitative real-time PCR

Murine BMDMs were harvested at various time points from the NT (non-treated) BMDM group or post-stimulation with *L. salivarius UCC118* at MOI (multiplicity of infection) of 10:1. Total RNA was extracted using the RNeasy Mini Kit (74104, Qiagen) followed by DNAse treatment using RNA Clean and Concentrator (R1015, ZYMO RESEARCH). cDNA was synthesized from total RNA as per manufacturers protocol using RT^2^ First Strand kit (330404, Qiagen). Expression profiling (RT-qPCR) of TLR pathway-associated genes was performed using RT^2^ Profiler PCR Array for Mouse TLR Signaling Pathway (PAMM- 018Z, Qiagen) at 20 h time point. Heatmaps were used to compare log2^−∆∆Ct^ values of *L. salivarius* UCC118 co-cultured BMDMs relative to NT. R library ggplot2 function was used to visualize the data in the form of heatmap. The data values ranges from − 1.69 to 4.53 and colored accordingly from blue to red. Separately, qRT-PCR (quantitative real-time PCR) for *Tlr1*, *Tlr2*, *Clec4e*, *Il6*, *Tnfa* and *Il1b* was performed at 4, 8, 12 and 20 h with primers and probes listed at Table 2. Data were normalized to average Ct values of *Actb* (beta-actin) and expressed as fold change (2^−∆∆Ct^).

**Table 2:** List of primers used in qRTPCR.

Primer	Sequence	UPL* probe no.
*Il6*_forward	5′-gctaccaaactggatataatcagga-3′	P6
*Il6*_reverse	5′-ccaggtagctatggtactccagaa-3′	P6
*Tnfα*_forward	5′-tcttctcattcctgcttgtgg-3′	P60
*Tnfα*_reverse	5′-ggtctgggccatagaactga-3′	P60
*Il1β*_forward	5′-agttgacggaccccaaaag-3′	P78
*Il1β*_reverse	5′-agctggatgctctcatcagg-3′	P78
*Tlr1*_forward	5′-ccagtggaagagactggactgt-3′	P2
*Tlr1*_reverse	5′-caactgcattcacaaacactca-3′	P2
*Tlr2*_forward	5′-ggggcttcacttctctgctt-3′	P50
*Tlr2*_reverse	5′-agcatcctctgagatttgacg-3′	P50
*Clec4e*_forward	5′-gcctccatcctgtttctcag-3′	P9
*Clec4e*_reverse	5′-ctgtaagttctgcccggaaa-3′	P9

*UPL* universal probe library.

### Flow cytometry analysis

BMDM cells were washed twice in PBS supplemented with 1% bovine serum albumin (BSA) and 0.1% sodium azide. Nonspecific binding of antibodies (Abs) to Fc receptors was blocked by pre-incubation of cells with monoclonal Abs (mAb) 2.4G2 directed against the FcgRIII/II CD16/CD32 (0.5 ng mAb per 10^6^ cells). 1 × 10^6^ cells were incubated with 0.5 ng of the relevant mAb for 20 min at 4 °C, and washed again twice. The following mAbs from ebiosicence were used: APC-conjugated mAb binding CD45, PECy-7-conjugated mAb binding CD11b, FITC-conjugated mAb binding F4/80 and PE-conjugated mAb binding TLR2. Cells were analysed using BD Accuri C6 or BD FACSCalibur. Data were analysed using FCS Express V5 Flow Cytometry software (Copyright De Novo Software 2017).

### Heat map generation of cytokine responses against bacterial strains

The log of the median of cytokine concentration (pg/ml) from three independent experiments for different bacterial strains was visualized in the form of heatmap (Fig. 1). The heatmap was generated using ComplexHeatMap R package^75^. The gradient of the heat maps generated goes from blue to red (on a range of − 5 to + 15) to depict the low, intermediate, or high cytokine secretion elicited by the test strains. Different environmental origins of *L. salivarius* strains are denoted by: a, animal; hb, human blood; hc, human intestine; hs, human saliva; un, unknown.

### Statistical analysis

Cytokine (IL-6, IL-10, IL-1β, IL-12p70, TNF-α and KC) responses from WT BMDMs treated with different ligands or co-cultured with different bacteria were compared with the cytokine response from TLR2^−/−^, TLR4^−/−^, TLR2/4^−/−^ or Clec4e^−/−^ BMDMs. TNF-α response and NF-κB reporter activity of THP1-XBlue cells (thpx-sp) were compared with THP1-XBlue- defMyD cells (thpx-dmyd). All experiments were repeated three times (n = 3) in triplicate wells per experiment. Statistical analysis were performed using 2 tailed Student T test in GraphPad Prism. p < 0.05 was considered as statistically significant.

## Supplementary Information

Supplementary Information 1.

Supplementary Information 2.

Supplementary Legends.

## References

[1] Gill SR (2006). Metagenomic analysis of the human distal gut microbiome. Science.

[2] Claesson MJ (2011). Composition, variability, and temporal stability of the intestinal microbiota of the elderly. Proc. Natl. Acad. Sci. USA.

[3] Smelt, M.J. *et al.**L. plantarum*, L. salivarius, and L. lactis attenuate Th2 responses and increase Treg frequencies in healthy mice in a strain dependent manner. *PLoS One* **7**, e47244 (2012). 10.1371/journal.pone.0047244 PMC3467239 23056616

[4] Liu X (2016). *Lactobacillus salivarius* isolated from patients with rheumatoid arthritis suppresses collagen-induced arthritis and increases treg frequency in mice. J. Interferon Cytokine Res..

[5] Peran, L. *et al.* Preventative effects of a probiotic, *Lactobacillus salivarius* ssp. salivarius, in the TNBS model of rat colitis. *World J. Gastroenterol.* **11**, 5185–5192 (2005). 10.3748/wjg.v11.i33.5185 PMC4320393 16127750

[6] de Andres, J. *et al.* Physiological translocation of lactic acid bacteria during pregnancy contributes to the composition of the milk microbiota in mice. *Nutrients* **10** (2017). 10.3390/nu10010014 PMC5793242 29295502

[7] O'Hara AM (2006). Functional modulation of human intestinal epithelial cell responses by *Bifidobacterium infantis* and *Lactobacillus salivarius*. Immunology.

[8] Panpetch W, Spinler JK, Versalovic J, Tumwasorn S (2016). Characterization of Lactobacillus salivarius strains B37 and B60 capable of inhibiting IL-8 production in *Helicobacter pylori*-stimulated gastric epithelial cells. BMC Microbiol..

[9] Dunne, C. *et al.* Probiotics: from myth to reality. Demonstration of functionality in animal models of disease and in human clinical trials. *Antonie Van Leeuwenhoek* **76**, 279–292 (1999). 10532384

[10] Corr SC (2007). Bacteriocin production as a mechanism for the antiinfective activity of *Lactobacillus salivarius* UCC118. Proc. Natl. Acad. Sci. USA.

[11] O'Mahony L (2001). Probiotic impact on microbial flora, inflammation and tumour development in IL-10 knockout mice. Aliment. Pharmacol. Ther..

[12] Miyauchi E (2012). Mechanism of protection of transepithelial barrier function by *Lactobacillus salivarius*: strain dependence and attenuation by bacteriocin production. Am. J. Physiol. Gastrointest. Liver Physiol..

[13] Ryan KA, O'Hara AM, van Pijkeren JP, Douillard FP, O'Toole PW (2009). Lactobacillus salivarius modulates cytokine induction and virulence factor gene expression in *Helicobacter pylori*. J. Med. Microbiol..

[14] Claesson MJ (2006). Multireplicon genome architecture of *Lactobacillus salivarius*. Proc. Natl. Acad. Sci. USA.

[15] Flynn, S. *et al.* Characterization of the genetic locus responsible for the production of ABP-118, a novel bacteriocin produced by the probiotic bacterium *Lactobacillus salivarius* subsp. salivarius UCC118. *Microbiology* **148**, 973–984 (2002). 10.1099/00221287-148-4-973 11932444

[16] O'Callaghan J, Butto LF, MacSharry J, Nally K, O'Toole PW (2012). Influence of adhesion and bacteriocin production by *Lactobacillus salivarius* on the intestinal epithelial cell transcriptional response. Appl. Environ. Microbiol..

[17] Kawai T, Akira S (2010). The role of pattern-recognition receptors in innate immunity: update on Toll-like receptors. Nat. Immunol..

[18] Harris G, KuoLee R, Chen W (2006). Role of Toll-like receptors in health and diseases of gastrointestinal tract. World. J. Gastroenterol..

[19] Kawai T, Akira S (2011). Toll-like receptors and their crosstalk with other innate receptors in infection and immunity. Immunity.

[20] Werling D, Jungi TW (2003). TOLL-like receptors linking innate and adaptive immune response. Vet. Immunol. Immunopathol..

[21] Zhu G (2018). Targeting pattern-recognition receptors to discover new small molecule immune modulators. Eur. J. Med. Chem..

[22] Torres D (2004). Toll-like receptor 2 is required for optimal control of Listeria monocytogenes infection. Infect. Immun..

[23] Hajishengallis G, Wang M, Bagby GJ, Nelson S (2008). Importance of TLR2 in early innate immune response to acute pulmonary infection with *Porphyromonas gingivalis* in mice. J. Immunol..

[24] Arias MA (2016). Toll-like receptors 2 and 4 cooperate in the control of the emerging pathogen *Brucella microti*. Front. Cell Infect. Microbiol..

[25] Arpaia N (2011). TLR signaling is required for *Salmonella typhimurium* virulence. Cell.

[26] Round JL, Mazmanian SK (2009). The gut microbiota shapes intestinal immune responses during health and disease. Nat. Rev. Immunol..

[27] Chassaing, B., Ley, R.E. & Gewirtz, A.T. Intestinal epithelial cell toll-like receptor 5 regulates the intestinal microbiota to prevent low-grade inflammation and metabolic syndrome in mice. *Gastroenterology* **147**, 1363–1377 e1317 (2014). 10.1053/j.gastro.2014.08.033 PMC4253564 25172014

[28] Fulde M (2018). Neonatal selection by Toll-like receptor 5 influences long-term gut microbiota composition. Nature.

[29] Vaishnava S (2011). The antibacterial lectin RegIIIgamma promotes the spatial segregation of microbiota and host in the intestine. Science.

[30] Round JL (2011). The Toll-like receptor 2 pathway establishes colonization by a commensal of the human microbiota. Science.

[31] Kondo T, Kawai T, Akira S (2012). Dissecting negative regulation of Toll-like receptor signaling. Trends Immunol..

[32] Leifer CA, Medvedev AE (2016). Molecular mechanisms of regulation of Toll-like receptor signaling. J. Leukoc. Biol..

[33] Takeuchi O (1999). Differential roles of TLR2 and TLR4 in recognition of gram-negative and gram-positive bacterial cell wall components. Immunity.

[34] Rikio Yabe, Y.I., Shinobu Sayo. C-type lectin receptors in host defense against microbial pathogens. Springer, Japan, 1319–1329 (2015).

[35] Fanaro S, Chierici R, Guerrini P, Vigi V (2003). Intestinal microflora in early infancy: Composition and development. Acta Paediatr. Suppl.

[36] Turroni F, van Sinderen D, Ventura M (2009). Bifidobacteria: From ecology to genomics. Front. Biosci. (Landmark Ed).

[37] Fanning S, Hall LJ, van Sinderen D (2012). Bifidobacterium breve UCC2003 surface exopolysaccharide production is a beneficial trait mediating commensal-host interaction through immune modulation and pathogen protection. Gut Microbes.

[38] Matsunaga N, Tsuchimori N, Matsumoto T, Ii M (2011). TAK-242 (resatorvid), a small-molecule inhibitor of Toll-like receptor (TLR) 4 signaling, binds selectively to TLR4 and interferes with interactions between TLR4 and its adaptor molecules. Mol. Pharmacol..

[39] Branger J (2004). Role of Toll-like receptor 4 in gram-positive and gram-negative pneumonia in mice. Infect. Immun..

[40] Jones SE, Paynich ML, Kearns DB, Knight KL (2014). Protection from intestinal inflammation by bacterial exopolysaccharides. J. Immunol..

[41] Laino, J., Villena, J., Kanmani, P. & Kitazawa, H. Immunoregulatory effects triggered by lactic acid bacteria exopolysaccharides: new insights into molecular interactions with host cells. *Microorganisms* **4** (2016). 10.3390/microorganisms4030027 PMC5039587 27681921

[42] Li M, Lin F, Lin Y, Peng W (2015). Extracellular polysaccharide from Bordetella species reduces high glucose-induced macrophage apoptosis via regulating interaction between caveolin-1 and TLR4. Biochem. Biophys. Res. Commun..

[43] Raftis EJ, Salvetti E, Torriani S, Felis GE, O'Toole PW (2011). Genomic diversity of *Lactobacillus salivarius*. Appl. Environ. Microbiol..

[44] Castro-Bravo N, Wells JM, Margolles A, Ruas-Madiedo P (2018). Interactions of surface exopolysaccharides from bifidobacterium and lactobacillus within the intestinal environment. Front. Microbiol..

[45] Ishikawa E (2009). Direct recognition of the mycobacterial glycolipid, trehalose dimycolate, by C-type lectin Mincle. J. Exp. Med..

[46] Bhatt K, Salgame P (2007). Host innate immune response to *Mycobacterium tuberculosis*. J. Clin. Immunol..

[47] Szklarczyk D (2019). STRING v11: Protein-protein association networks with increased coverage, supporting functional discovery in genome-wide experimental datasets. Nucleic Acids Res..

[48] Akira S, Hemmi H (2003). Recognition of pathogen-associated molecular patterns by TLR family. Immunol. Lett..

[49] Zegarra-Ruiz, D.F. *et al.* A diet-sensitive commensal lactobacillus strain mediates TLR7-dependent systemic autoimmunity. *Cell Host Microbe* **25**, 113–127 e116 (2019). 10.1016/j.chom.2018.11.009 PMC6377154 30581114

[50] Hiramatsu Y (2019). Lactobacillus plantarum induces genomic DNA-dependent and TLR9-mediated elafin secretion from Caco-2 cells. Asian Pac. J. Allergy Immunol..

[51] Jiang Z (2003). Poly(I-C)-induced Toll-like receptor 3 (TLR3)-mediated activation of NFkappa B and MAP kinase is through an interleukin-1 receptor-associated kinase (IRAK)-independent pathway employing the signaling components TLR3-TRAF6-TAK1-TAB2-PKR. J. Biol. Chem..

[52] Park JH (2007). RICK/RIP2 mediates innate immune responses induced through Nod1 and Nod2 but not TLRs. J. Immunol..

[53] Adachi O (1998). Targeted disruption of the MyD88 gene results in loss of IL-1- and IL-18-mediated function. Immunity.

[54] Medzhitov R (1998). MyD88 is an adaptor protein in the hToll/IL-1 receptor family signaling pathways. Mol. Cell.

[55] Gasse P (2007). IL-1R1/MyD88 signaling and the inflammasome are essential in pulmonary inflammation and fibrosis in mice. J. Clin. Invest..

[56] Peres AG (2015). Uncoupling of pro- and anti-inflammatory properties of Staphylococcus aureus. Infect Immun.

[57] Koch BEV, Yang S, Lamers G, Stougaard J, Spaink HP (2018). Intestinal microbiome adjusts the innate immune setpoint during colonization through negative regulation of MyD88. Nat. Commun..

[58] Abdollahi-Roodsaz S (2013). Toll-like receptor 2 controls acute immune complex-driven arthritis in mice by regulating the inhibitory Fcgamma receptor IIB. Arthritis Rheum.

[59] Ey B (2013). Loss of TLR2 worsens spontaneous colitis in MDR1A deficiency through commensally induced pyroptosis. J. Immunol..

[60] Chang YC (2017). TLR2 and interleukin-10 are involved in Bacteroides fragilis-mediated prevention of DSS-induced colitis in gnotobiotic mice. PLoS ONE.

[61] Olfe J, Domanska G, Schuett C, Kiank C (2010). Different stress-related phenotypes of BALB/c mice from in-house or vendor: Alterations of the sympathetic and HPA axis responsiveness. BMC Physiol..

[62] Sellers RS, Clifford CB, Treuting PM, Brayton C (2012). Immunological variation between inbred laboratory mouse strains: Points to consider in phenotyping genetically immunomodified mice. Vet. Pathol..

[63] Becker C, Neurath MF, Wirtz S (2015). The intestinal microbiota in inflammatory bowel disease. ILAR J..

[64] Kamdar K (2018). Innate recognition of the microbiota by TLR1 promotes epithelial homeostasis and prevents chronic inflammation. J. Immunol..

[65] Vaishnava S, Behrendt CL, Ismail AS, Eckmann L, Hooper LV (2008). Paneth cells directly sense gut commensals and maintain homeostasis at the intestinal host-microbial interface. Proc. Natl. Acad. Sci. USA.

[66] Hoving JC, Wilson GJ, Brown GD (2014). Signalling C-type lectin receptors, microbial recognition and immunity. Cell Microbiol..

[67] Wells CA (2008). The macrophage-inducible C-type lectin, mincle, is an essential component of the innate immune response to *Candida albicans*. J. Immunol..

[68] Schick, J. *et al.* Toll-like receptor 2 and mincle cooperatively sense corynebacterial cell wall glycolipids. *Infect. Immun.* **85** (2017). 10.1128/IAI.00075-17 PMC5478951 28483856

[69] Shah S, Nagata M, Yamasaki S, Williams SJ (2016). Total synthesis of a cyclopropane-fatty acid alpha-glucosyl diglyceride from *Lactobacillus plantarum* and identification of its ability to signal through Mincle. Chem. Commun. (Camb).

[70] Martinez-Lopez, M. *et al.* Microbiota sensing by Mincle-Syk axis in dendritic cells regulates interleukin-17 and -22 production and promotes intestinal barrier integrity. *Immunity* **50**, 446–461 e449 (2019). 10.1016/j.immuni.2018.12.020 PMC6382412 30709742

[71] Pahari S (2020). Induction of autophagy through CLEC4E in combination with TLR4: An innovative strategy to restrict the survival of Mycobacterium tuberculosis. Autophagy.

[72] Kelly P (2005). Correlation of probiotic Lactobacillus salivarius growth phase with its cell wall-associated proteome. FEMS Microbiol. Lett..

[73] Sengupta R (2013). The role of cell surface architecture of lactobacilli in host-microbe interactions in the gastrointestinal tract. Mediat. Inflamm..

[74] Weischenfeldt, J. & Porse, B. Bone Marrow-Derived Macrophages (BMM): Isolation and Applications. *CSH Protoc* **2008**, pdb prot5080 (2008). 10.1101/pdb.prot5080 21356739

[75] Gu Z, Eils R, Schlesner M (2016). Complex hea®aps reveal patterns and correlations in multidimensional genomic data. Bioinformatics.

