# Supplementary figures and images for: Macrophage cytokine responses to commensal Gram-positive Lactobacillus salivarius strains are TLR2-independent and Myd88-dependent
Source: Sci Rep. 2021 Mar 15;11:5896. doi: 10.1038/s41598-021-85347-7 (PMC7961041; doi:10.1038/s41598-021-85347-7)

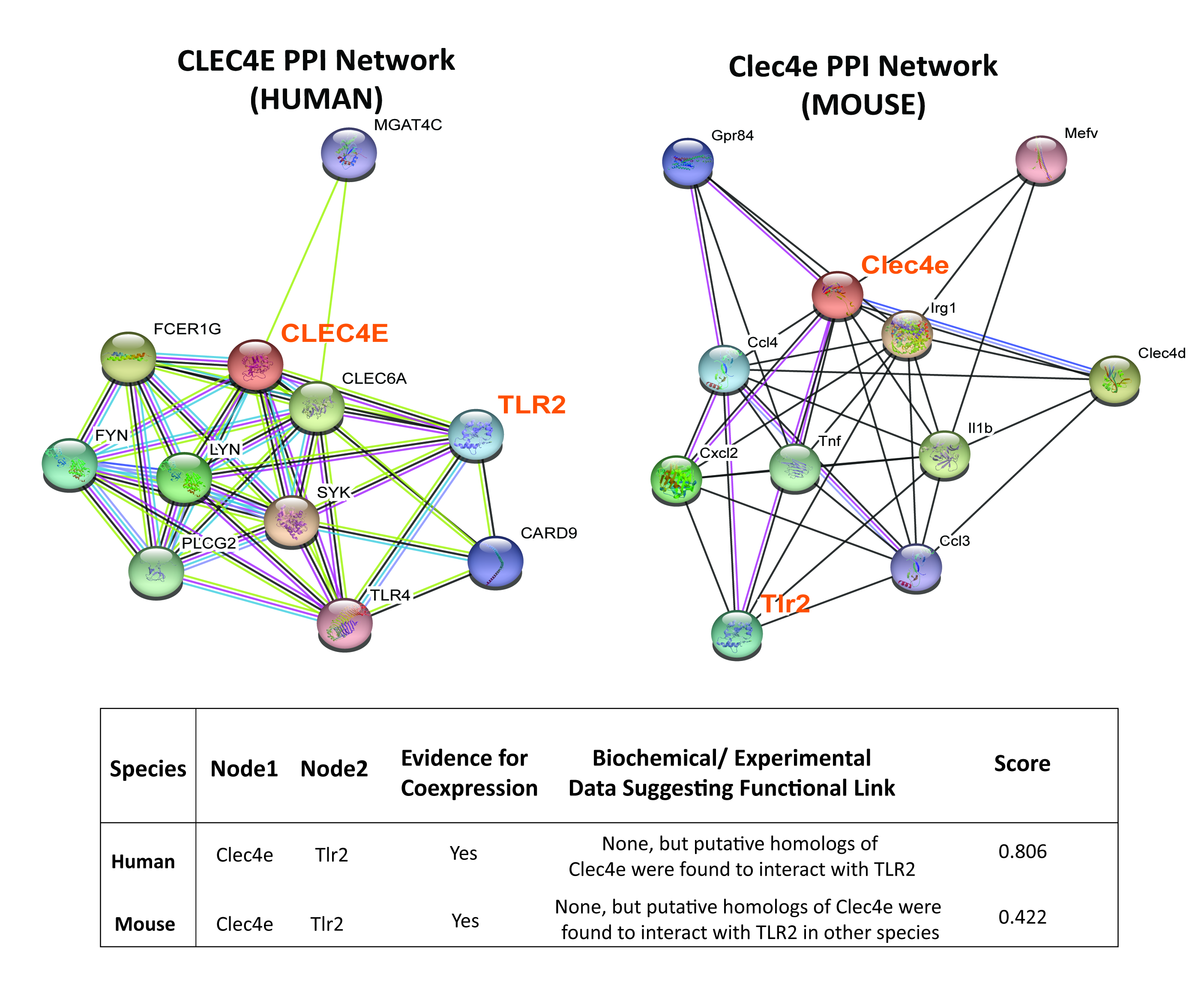

Supplement: Supplementary file 1 — Supplementary Information 1. [file 41598_2021_85347_MOESM1_ESM.tif]

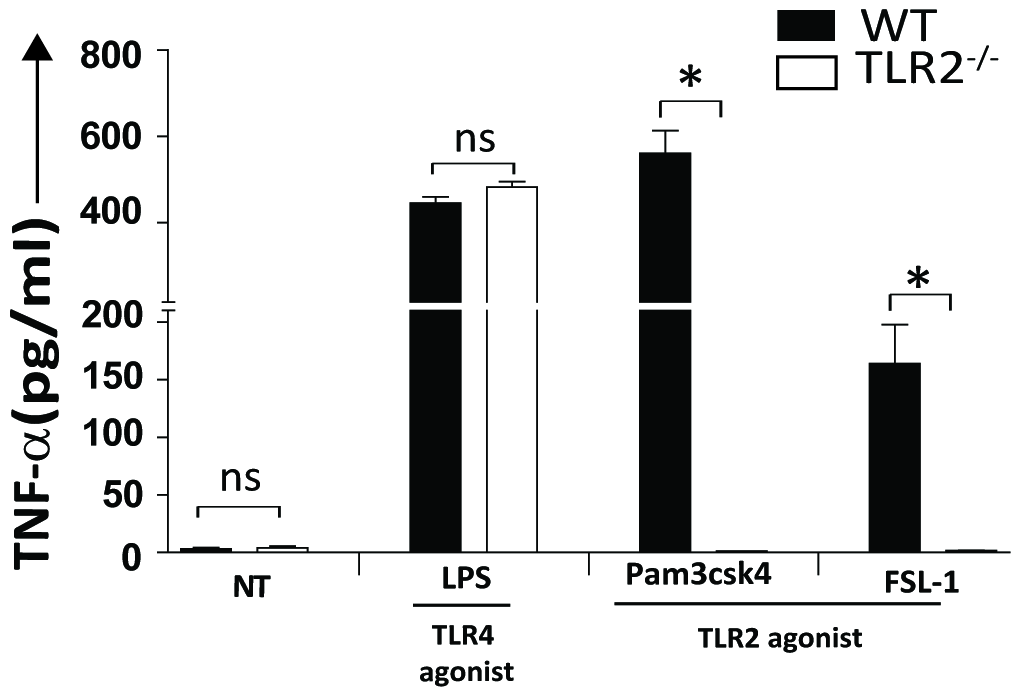

Supplement: Supplementary file 2 — Supplementary Information 2. [file 41598_2021_85347_MOESM2_ESM.tif]
