# Supplementary material for: Macrophage cytokine responses to commensal Gram-positive Lactobacillus salivarius strains are TLR2-independent and Myd88-dependent
Source: Sci Rep. 2021 Mar 15;11:5896. doi: 10.1038/s41598-021-85347-7 (PMC7961041; doi:10.1038/s41598-021-85347-7)
Supplement: Supplementary file 3 — Supplementary Legends. [file 41598_2021_85347_MOESM3_ESM.docx]

**Supplementary Figure 1** *LPS induced cytokine responses are TLR2 independent but FSL-1 and Pam3csk4 induced cytokine responses are TLR2 dependent in BMDMs.*

TNF-α cytokine secretion (pg/ml) was quantified by ELISA from WT and TLR2^-/-^ BMDMs that were non treated (NT) or treated with TLR1/2 agonist (Pam3csk4), TLR2/6 agonist (FSL-1) and TLR4 agonist (LPS) for 20 hours.. Data shown are the average of triplicate wells of three independent experiments (n=3). Data are the average of three independent experiments (n=3). Statistical analysis was performed with 2 tailed student t test in GraphPad Prism, p<0.05 (denoted by *) was considered statistically significant. ns- nonsignificant

**Supplementary Figure 2** *STRING protein-protein interaction database identifies co-expression and functional association of Mincle protein family members with TLR2 in mouse and humans.*

STRING database was searched for proteins that are co-expressed or functionally associated with Clec4e (Mincle) at score>0.4 in mouse and humans. Score defines the approximate confidence, on a scale of zero to one, of the association being true, given all the available evidence.
